# Editorial: Immunity to Fungal Infections: Insights From the Innate Immune Recognition and Antifungal Effector Mechanisms

**DOI:** 10.3389/fmicb.2021.714013

**Published:** 2021-07-15

**Authors:** David L. Moyes, Allan J. Guimarães, Rodrigo T. Figueiredo

**Affiliations:** ^1^Centre for Host-Microbiome Interactions, Faculty of Dentistry, Oral and Craniofacial Sciences, King's College London, London, United Kingdom; ^2^Department of Microbiology and Parasitology, Biomedical Institute, Fluminense Federal University, Niterói, Brazil; ^3^Campus Duque de Caxias, Federal University of Rio de Janeiro, Duque de Caxias, Brazil

**Keywords:** mycoses, innate immunity, leukocytes, lymphocytes, cytokines

Fungal infections represent a major health concern which, according estimations, account for nearly 1.6 million deaths annually (Brown et al., 2012). Epidemiological reports have demonstrated a growth in the incidence of invasive mycoses which are associated with conditions associated with immunossupression, such as diabetes, immunosupressive therapy for solid organ transplantation or autoimmune diseases, cancer and neutropenia (Bitar et al., 2014; Rayens et al., 2021). Risk factors for invasive mycosis represent medical advances, including the use of intravenous catheters, cancer chemotherapy and immunosupressive therapies which have created a growing population of vulnerable patients. Invasive mycoses are associated with unacceptable high lethality rates (Suleyman and Alangaden, 2016). Although antifungal drug resistance is not a major problem in most cases of invasive mycoses, it is an additional threat (Fairlamb et al., 2016). Recent outbreaks of infections caused by *Candida auris*, a multidrug-resistant fungus, as well as associations of fungal infections with influenza and COVID-19 cases, as observed for aspergillosis and mucormycosis, have created new challenges for the managements of patients in critical care units (Spivak and Hanson, 2018; Dewi et al., 2021; Gandra et al., 2021).

Integration of innate immune recognition and adaptive immunity is critical for the control of invasive mycoses. The relevance of the basic knowledge in the immunopathogenesis of mycoses can be observed by the growth in clinical immunomodulation studies (Williams et al., 2020). Thus, comprehension of the immunity to fungal pathogens offers new possibilities for the treatment of invasive mycoses. This topic discusses aspects of the innate immunity and leukocyte activation to fungal pathogens, the repertoire of B and T cell antigen receptors in experimental models of pneumocystosis, as well as a discussion about the control of the expression of fungal toxins involved in the modulation of host responses.

Here, Thompson et al. investigate the role of Dectin-2 and Mincle in recognizing different *Candida* species—notably *C. albicans, C. parapsilosis, C. tropicalis*, and *C. glabrata*. They compare the cell wall composition of the different species, looking at cell wall thickness and phosphomannan content, and discuss the importance of Mincle and Dectin-2 recognition in systemic murine infection models varies with the *Candida* species, indicating the varied nature of fungal detection in the innate immune response.

Neutrophil DNA extracellular traps (NETs) are formed in response to fungal pathogens and show fungicidal/fungistatic activity (Urban and Nett, 2019). Eosinophils ETs (EETs) have been observed in human samples from bronchopulmonary aspergillosis cases and human eosinophils release ETs in response to *A. fumigatus* conidia (Muniz et al., 2018). In this topic, Silva et al. present a review discussing the mechanisms involved in the release of NETs and EETs, in response to fungal pathogens, as well as the role of ETs in the immunity and pathogenesis of fungal infections. In this issue, Barroso et al. describe the signaling pathways involved in the EET formation in response to *A. fumigatus*. *A. fumigatus*-induced EET release requires the activity of Src kinases, class IA phosphatidylinositol 3-kinase δ (PI3KIδ), Akt and p38 kinase. Intracellular calcium mobilization is also required for EET formation in response to *A. fumigatus*, while PAD4-mediated histone citrullination is dispensable. EET release does not require *A. fumigatus* viability, indicating that recognition of *A. fumigatus* molecular patterns is able to trigger EETosis, while fungal viability and expression of virulence factors are dispensable for the eosinophil activation culminating in the ET release.

Fungal infections are a particular problem in HIV-infected individuals. Indeed, one of the defining features of AIDS is respiratory *Pneumocystis* infection. Normally, these infections are prevented by robust B and T cell responses (Thomas and Limper, 2007). Although gross examination of the different subsets of T cells during these infections has been identified, only recently the tools have been developed to increase the resolution of the T cell immunophenotypes. In this issue, Yang et al. use a combination single cell TCR-Seq and single cell RNA-Seq to identify the subsets of T cells that respond to *Pneumocystis* infection. In doing so, they reveal the composition and characteristics of clonally expanding T cells, along with their TCR repertoire.

In a similar approach, Sun et al. integrated single-cell RNA and BCR sequencing of immune cells from mouse lungs to detail the dynamic nature of B cell responses during Pneumocystis infection, with ongoing increased plasma cells elevated ratio of (IgA+ IgG) to (IgD+ IgM) after infection. Despite the clonal expansion post-infection, BCR repertoire diversity decreased, with B cell transcriptional changes including a biased usage of V(D)J genes and higher frequency of somatic hypermutation in comparison to naïve B cells, offering valuable information and tools for the development of immunotherapeutic targets and diagnostic biomarkers.

The modulation of inflammatory responses during fungal infections, particularly in the context of how each IL-1 family (IL-1, IL-18, and IL-36) member, despite of their clearly defined roles, could act together in determining the disease outcome is reviewed by Griffiths et al.. The authors address their mechanism of induction, the main cellular types responsible for their expression and processing and the immunological and other functional roles of each subfamily member. The authors tackle deeply how several endemically important fungal pathogens could differentially induce each of these IL-1 subfamily cytokines. Their therapeutic potential is further described as modulation of receptors or proteins regulating the IL-1 subfamily might enhance protective anti-fungal immunity or resolve excessive damaging immune responses.

Host and fungus interactions trigger responses in a two-way signaling that leads to fungal adaptation to the host environment, for example the hyphal growth in *Candida* species. Notably, these interactions can lead to the secretion of different mycotoxins, including ochratoxin, which can mediate different aspects of the pathology associated with infection. In this issue, Gao et al. review the role of G protein coupled receptors (GCPR) in fungi, and their role in regulating the synthesis of mycotoxins. Accordingly, the authors explore the current limited state of knowledge of fungal GPCR biology and how these circuits regulate fungal behavior and mycotoxin synthesis. The role of GPCR as potential targets in modulating the synthesis of secondary fungal metabolites and regulating fungal behavior is also discussed.

## Author Contributions

All authors contributed to manuscript revision and draft, read, and approved the submitted version.

## Conflict of Interest

The authors declare that the research was conducted in the absence of any commercial or financial relationships that could be construed as a potential conflict of interest.
